# How changes in GPs’ ways of working have affected community nurses: a qualitative study

**DOI:** 10.3399/BJGP.2024.0534

**Published:** 2025-05-07

**Authors:** Louisa Polak, Kristian Pollock, Stephen Barclay, Ben Bowers

**Affiliations:** Department of Public Health and Primary Care, University of Cambridge, Cambridge.; School of Health Sciences, University of Nottingham, Nottingham.; Department of Public Health and Primary Care, University of Cambridge, Cambridge.; Department of Public Health and Primary Care, University of Cambridge, Cambridge; honorary associate professor, School of Health Sciences, University of Nottingham, Nottingham.

**Keywords:** community health nursing, community paramedics, general practice, palliative care, remote working, task-shifting

## Abstract

**Background:**

A growing literature examines the way two changes in primary care — the shift towards remote working and the diversification of practice teams to incorporate, for instance, physician associates and paramedics — affect patient care within the practice. However, little is known about the effect of these changes on community nurses.

**Aim:**

To explore community nurses’ experiences of delivering palliative care in the context of GPs’ new ways of working.

**Design and setting:**

Qualitative study using focus groups in the UK.

**Method:**

Focus groups were conducted on Zoom with community nurses. Data were analysed thematically using constant comparison.

**Results:**

Community nurses described extending their roles in palliative care. Alongside pride and satisfaction about this, participants raised several areas of concern and dissatisfaction, some of which they associated with changes in GPs’ ways of working. Two reasons for dissatisfaction concerned remote working. First, remote communication with colleagues was seen as creating obstacles to nurses’ everyday collaboration with GPs, damaging important working relationships. Second, nurses increased their workload by taking the lead in person-centred care where they saw remote provision by GPs as unsatisfactory. Where workforce diversification led to delegating home visits to paramedics or nurse practitioners, community nurses described feeling a lack of the ‘GP back-up’ that many identified as essential for community palliative care.

**Conclusion:**

When considering and evaluating interventions that change the way GPs work, policymakers and commissioners should look not only at consequences that affect primary care teams, but also at the effects across the complex ecosystem within which these teams operate.

## Introduction

The way GPs deliver care has changed in two significant ways recently: more work is done remotely and the workforce has become more diverse. A growing body of research^1^^,^^2^ has examined the effects of these changes on clinicians and patients. However, the effects on healthcare providers who routinely collaborate with primary care teams have not yet, to our knowledge, been studied; we offer an account of community nurses’ perceptions of these effects within the context of palliative care.

Community or ‘district’ nurses work closely with GPs to care for patients who are housebound, although they are funded and managed as a separate service within the UK NHS. The importance of close collaboration between these different bodies of professionals has been identified^3^ in the context of caring for patients who are older, one of the groups most likely to be housebound; the other (overlapping) big group requiring care at home comprises those approaching the end of life. When adequate care cannot be provided remotely, patients who are housebound need home visits by clinicians. Meeting this need may be challenging for increasingly stretched GPs. A UK-based review^4^ has highlighted calls to remove home visiting from the list of ‘core services’ GPs are contracted to offer, and the review examined the effects on service provision of the two moves we consider here: using virtual consultations and shifting the task of undertaking home visits to other clinicians.

Palliative care, the lens for our research, has long constituted a significant element of community nurses’ work,^5^ where it has grown in prominence as the number of people dying at home has increased.^6^ Policy imperatives to minimise hospital admissions towards the end of life, in response to cost pressures and perceived patient preference for home care,^7^ reinforce the importance of community palliative care.

In our study, community nurse participants were invited to talk about their experiences of providing palliative care, particularly about ways in which their roles had changed in recent years. Although our data come from interviews with community nurses, they are highly relevant to the GPs working alongside them, and have implications for policymakers considering making further changes to the way GPs work.

**Table 1:** How this fits in

Increased remote working and task-shifting to other primary care clinicians are two significant changes in the way GPs work. When invited to talk about collaborating with GPs to deliver palliative care, community nurses described extending their roles, taking more responsibility for person-centred care including ‘difficult’ conversations and complex decisions. This shift was seen as having mixed positive and negative effects on the experience of community nursing, and as being driven at least partly by GPs’ new ways of working. Our findings thus complement the growing literature about the effects these new ways of working have on primary care teams and their patients, highlighting the importance of looking at consequences across the whole ecosystem of community care provision when planning or evaluating changes within general practice.

## Method

This qualitative study of community nurses’ experiences of providing palliative care used focus groups, allowing participant interactions to be used alongside individual accounts as a source of analytic purchase.^8^

### Recruitment

Participants were registered nurses working in UK community nursing teams. All had provided end-of-life care for adult patients in the community within the past 3 months. From 51 nurses who took part in a preliminary descriptive questionnaire about their changing roles in palliative care since the start of the COVID-19 pandemic, 35 focus group participants were recruited purposively to ensure a spread of geographical areas, pay bands, and years of experience. Supplementary Box S1 summarises recruitment strategies for the preliminary questionnaire. Supplementary Table S1 provides demographic information about focus group participants.

### Data collection

Data were collected during March and April 2023. Ten focus group interviews were conducted via Zoom by an experienced researcher.

The topic guide (see Supplementary Information S1) was constructed and refined in consultation with a small advisory group: two family carers and two community nurses. This guide explored how community nurses’ roles had changed since the start of the COVID-19 pandemic. It was used in a semi-structured way, using the listed questions as prompts from which group conversations were allowed to evolve.

Data were transcribed verbatim and anonymised.

### Analysis

Two authors (the first author and the senior author) read all transcripts, discussed and refined their respective line-by-line coding, and undertook thematic analysis to produce a thick ‘qualitative description’.^9^ One salient theme within this description was community nurses’ perceptions of the ways in which their roles were affected by GPs’ new ways of working. The first author and the senior author along with the second author then used constant comparison^10^ to build a detailed, theoretically informed picture of what was going on. Comparing participants’ differing accounts with one another, as well as with the relevant literature, we paid particular attention to instances where what one participant said was at odds with most others. Analytic rigour was reinforced by discussing findings and provisional explanations with an additional author (the third author), and within the primary care research group to which the authors belong.

## Results

Participants had been invited to talk about changes to their own roles in community palliative care. Unexpectedly, they talked a lot about their interactions with GPs, and it is their accounts of these interactions that we draw on here.

The main new roles mentioned were verifying death and prescribing; GPs were mentioned indirectly in connection with these, where community nurses described being expected to take on new tasks — *‘extra things keep being added on’* (Group 3) — which participants said used to be done by GPs:

*‘The GPs seem more remote now and we seem very heavily involved* […] *it’s totally switched round really.’*

(Group 4).

Alongside these accounts of new tasks, a more complex picture emerged when nurses talked about ways in which their established roles had extended and deepened. It is this picture that we focus on here. Within it, we highlight accounts of GPs’ new ways of working and their perceived effects on community nurses’ work. Two changes, increased remote working by GPs and diversification within primary healthcare teams, were both seen as problematic, particularly where community nurses sought to collaborate in caring for people with complex problems at the end of life. Underpinning the data we present here about these two issues is the assumption, widely visible in participants’ accounts, that:

*‘An important part of nursing at home is that we need the GP back-up.’*

(Group 8)

### Perceived impact of remote working

Nurses identified GPs’ adoption of alternatives to face-to-face encounters as affecting them in two ways. First, moving to remote encounters with other healthcare professionals made it harder to build and maintain working relationships and to communicate quickly and effectively about a patient. Second, GPs moving away from face-to-face encounters with patients was felt to contribute to task-shifting from GPs to community nurses, helping drive the extension of nurses’ established roles.

#### Remote communication between colleagues.

There was considerable variation in participants’ accounts of their working relationships with GPs. Some had been able to go back to face-to-face communication (after a pause during the pandemic); the following exchange shows that this was regarded by both speakers as a good thing, although it was not considered universally possible:

Speaker 1: *‘We’re quite lucky because we’re quite rural* […] *So we do have good, I mean I’m in the same physical building as both the practices.’*

Speaker 2: *‘Wow!’*

Speaker 1: ‘[…] *so yeah we’re quite different* […] *I can walk around and grab somebody if I really needed to.’* (Group 2)

Further variation was visible where practices asked nurses to communicate remotely with GPs, either by using various messaging systems or else by phoning. Of practices who preferred to be telephoned, some set up professionals-only access, bypassing the patients’ line, but participants reported that others did not do this, leading to frustrating delays and a feeling of professional disrespect. Even where nurses were given a priority number, it linked to a receptionist:

*‘We have a direct line that bypasses the patient line but it’s still straight through to a receptionist, so you’re still then relying on the receptionist then agreeing to message the duty doctor* […] *they’re on their mobiles so the surgery then phones them and you’re then waiting for them to phone you back.’*

(Group 8)

As well as often being slow, getting the receptionist to agree to contact the doctor was felt to be an annoying obstacle to efficient communication, adding an unwelcome layer of gatekeeping:

*‘It’s so frustrating because you just think if you just put me through to the GP it would take me two minutes to explain it, whereas I’m trying to explain it to you who doesn’t understand* […] *but they’ve got that pressure from the GP not to put us through to them* […] *they get* […] *quite short with us, like well what’s this, I’m trying to speak, can I just speak to a GP, and they’re like, well no if you just tell me what it is and I’ll task them.’*

(Group 7)

These practical difficulties were seen as contributing to the weakening of interprofessional relationships:

*‘We’ve lost touch with our GPs* […] *the relationships aren’t as strong as they used to be.’*

(Group 6)

*‘I don’t know any of the GPs now, I very rarely speak to them, each one has a different way of communicating, emails, tasks, different systems to write on, the relationships are so fractured and the patients just say to you “I don’t know who my GP is”.’*

(Group 4)

#### Remote communication with patients.

As the previous excerpt illustrates, some participants suggested that GPs’ increased use of remote working was a factor leading patients to say they do not know who their GP is. These suggestions often led into nurses’ accounts of having to extend their previous roles to take the lead regarding person-centred palliative care of people with complex problems:

*‘You just naturally have to do* [difficult conversations about future care preferences] *because over the telephone you don’t have that same relationship.’*

(Group 3)

Video consultations were not perceived as better in this respect:

*‘The GPs in my area still do it all over video so they can’t really have that real person-centred care’.*

(Group 1)

Many nurses described pride and satisfaction at taking the lead in providing this care:

*‘We build up such good relationships with our patients, it kind of goes hand-in-hand with that relationship that we’ve established, and the trust.’*

(Group 3)

But some of the same participants expressed resentment about what they saw as a move from GP-led to nurse-led care:

*‘The GPs are sort of thinking, well they can do it so we don’t need to be involved, so it’s putting more pressure on us.’*

(Group 1)

In summary, participants saw the move towards remote working as deleterious both to GPs’ collaborative relationships with their community nursing colleagues and to their ability to provide person-centred care for patients and their families. This reduced ability was seen as contributing to the shifting of work from general practices to community nurses.

### Workforce diversification and task-shifting within practices

Our community nurse participants commonly expressed concern about workforce diversification within general practice. This concern centred on the shifting of tasks from GPs to other primary care clinicians, and was underpinned by the widely expressed feeling that community nurses want and need support with managing the complex needs of their patients who are receiving palliative care. Although a few participants mentioned getting this support from palliative care specialists, most emphasised the need for ‘GP back-up’. Accounts of seeking back-up varied between participants. Some spoke of routinely being offered a paramedic or nurse practitioner to speak with instead of a doctor when they asked for medical input from the practices they worked with:

*‘A lot of the housebound patients are visited by their paramedic practitioners or their advanced clinical practitioners from the surgeries.’*

(Group 8)

The participant quoted next compares two practices in their area, highlighting that some practices do still offer what was seen as adequate GP back-up. In so doing, the practice is seen as showing ‘respect’ for the community nurse’s assessment of the situation; implicitly, a lack of respect is indicated by either declining to visit or delegating the task to a paramedic:

*‘If myself or any of the staff nurses go one practice with an issue about a patient, like* [name of participant] *was saying if you’ve gone to a GP to ask a GP to review a patient it’s because you need a GP to review that patient, and they will respect that. The other practice don’t, and they would ask a paramedic to go sometimes, or they won’t visit.’*

(Group 2)

Concerns about lack of ‘respect’ were also voiced elsewhere in our data, in the context of professional colleagues’ perceived lack of acknowledgement of the scope and depth of community nurses’ competence.

Participants’ negative comments about paramedics were mainly rooted in a broad discomfort with the way traditional clinical hierarchies were being disrupted by diversification. This discomfort was sometimes visibly infused with a normative discourse that was critical of GPs and implicitly equated traditional with ‘normal’ or proper ways of working:

*‘The surgery I work for have got two paramedics who are absolutely fantastic but they are not GPs, and I think the GPs need to stop relying on other services and come in and actually muck in again, get their hands dirty and get back to some form of normality.’*

(Group 1)

The move away from this ‘normality’ was at odds with expectations based on the hierarchy that has traditionally placed doctors above nurses, with paramedics below community nurses or at most on the same ‘level’:

*‘If I’ve requested for a GP to go and see a patient I’ve seen, I would expect somebody senior to review that patient, not someone at my level.’*

(Group 2)

*‘They seem to be sending like paramedic practitioners instead and their kind of skill scope is quite similar to ours* […] *you request one thing* [that is a GP assessment] *and you kind of get another.’*

(Group 4)

In contrast with these negatively flavoured comments, other participants spoke positively about the new clinical roles within primary care. Rather than focusing on levels within a linear hierarchy, these nurses spoke of paramedics’ skills as complementing their own, particularly in relation to assessing and managing a patient’s acute deterioration and avoiding hospital admission:

*‘Admission avoidance is something that paramedics are quite good at, they’ll liaise with GP practices, with care agencies et cetera to try and keep patients at home if that’s the best place for them.’*

(Group 5)

Even where this positivity was visible, however, many participants indicated unease and unhappiness when talking about their extended role in assessing and managing complex problems. These community nurses spoke of being given more responsibility than they felt comfortable with. They noted that their pay had not increased to reflect this increased responsibility; that they felt anxious that they sometimes risked their professional registration by acting autonomously without GP input; and — referencing traditional norms again — that they were taking *‘clinical decisions that we perhaps* shouldn’t *be doing’* (Group 1, Roman indicates emphasis), work that they felt was part of *‘the duties of a GP’*:

*‘There’s no recognition in our pay that we’re taking on basically the duties of a GP* […] *we are just so autonomous and with that comes a massive responsibility* […] *We are putting our registration at risk quite often* […] *for patient care.’*

(Group 6)

Particular concern was expressed about the work of assessing a complex situation in order to make clinical decisions; participants spoke about ‘diagnosis’, uncertainty, and the worrying possibility of being ‘wrong’:

*‘Most nurses aren’t diagnosticians, it’s not part of our* […] *remit unless you’ve done additional training, so* [it’s hard] *for us to sort of go “oh this is what we think might be happening but we don’t know for sure”* […] *when you haven’t got the confirmation from a medical professional, a GP or a doctor who is meant to diagnose things* […] *There’s an overreliance on the community nurses especially to diagnose the problem and then the GPs will just react to whatever we’re telling them without actually seeing the patient face to face. And we might be wrong, you know, we might be wrong.’*

(Group 8)

Calling this *‘overreliance’* indicates the perception widely visible in these data that GPs’ task-shifting, both to other clinicians within the practice and to their community nursing colleagues, has led to some unwelcome changes in nurses’ roles, albeit also some changes that were welcomed by participants. Overall, community nurses’ accounts were often coloured by anxiety about complex decision making with what was perceived to be inadequate senior support, and by resentment at what they described as lack of recognition of their extended roles and absence of consultation about the changes.

## Discussion

### Summary

Our findings are summarised in Supplementary Figure S1. They provide a description of community nurses’ perceptions of two changes within primary care: remote working and workforce diversification. A common thread links concerns that participants raised about these two separate changes: both were seen as damaging the relationships between community nurses and GPs, and as making it harder for nurses to get the senior clinical support they want. Without this support, nurses reported that they had felt obliged to extend their role to cover aspects of care formerly the remit of a GP, taking a lead in building the relationships with patients and their families that enabled shared decision making about complex problems. Many expressed a wish for increased GP support with these difficult decisions, alongside a perception that their roles had been extended with inadequate consultation and attracted inadequate acknowledgement and remuneration.

### Strengths and limitations

An open-ended, exploratory approach to both data collection and analysis was a strength of the study, helping generate a nuanced picture that represented the diversity of participants’ accounts. This approach enabled us to shed light on participants’ perceptions of changes in general practice, a topic that was not a focus of the initial research question.

Data collection would have been enriched by having a second researcher present during all focus groups and contributing their reflections; we only did this in four sessions, and the observers’ memos (directed primarily at providing formative feedback to the facilitator) were not used in the analysis.

The transferability of our findings to other times and places is limited by the study’s timing: we should assume that some effects of the COVID-19 pandemic were still evolving in 2023, a year after the last lockdown ended in the UK, so a later study may well yield different results.

Our findings report nurses’ accounts of their experiences. They should not be used as a basis for inferences about what GPs do in practice.

### Comparison with existing literature

In relation to remote working, the move to replace many face-to-face encounters with remote ones has been controversial in both public debate and academic discussions concerning a wide range of contexts.^11^ That this move was accelerated by the COVID-19 pandemic^12^ is clearly illustrated in the context of health care: caveats about limited usefulness and technological challenges in 2019^13^^,^^14^ contrast with enthusiastic endorsements published after the pandemic^15^ and implicit in the 2023 NHS Long Term Workforce Plan’s focus on telemedicine.^16^

However, a recent international review of remote consulting in primary care^1^ warned that face-to-face consultations remained important for *‘dealing with multiple issues and providing complex care’*, a warning clearly applicable to community palliative care. Echoing this, our participants expressed dissatisfaction with GPs who deliver such care remotely, and described feeling compelled to lead on providing person-centred care because they believe GPs cannot do so adequately ‘over video’.

Most research and guidance about remote working in health care centres on clinicians’ encounters with patients, but our findings highlight a second focus of concern: remote interactions replacing face-to-face conversations between clinicians. Visit requests, which have long been a site of difficult negotiations between district nurses and GPs,^17^ were a particular focus of participants’ annoyance. Their dissatisfactions about interactions with GPs stemmed both from remote working and from workforce diversification: irritation about having to use a variety of often cumbersome messaging technologies and telephone systems to communicate with GPs was compounded by being offered a paramedic or advanced nurse practitioner to talk with, rather than a GP. Additionally, nurses bemoaned the increasing rarity of opportunities to undertake visits jointly with a GP. Research about interprofessional working^18^ has emphasised the value of joint visits in establishing beneficial ‘role overlap’. Such overlap, and the shifting role boundaries that contextualise it, are important features of the changing ecosystem of community healthcare provision, brought into prominence by increasing role diversification and delegation within general practices.

In relation to workforce diversification, unlike remote working, this was not accelerated by the COVID-19 pandemic. In the UK, for example, the Additional Roles Reimbursement Scheme began to promote the development of new clinical roles within general practice in 2019.^19^ This intervention has become a focus of particularly intense scrutiny and debate^20^ that focuses predominantly on the potential adverse effects on GPs and patients,^21^ leaving a knowledge gap regarding possible effects on community nurses.

The chief aim of workforce diversification, and its close relatives ‘skill mix’ and ‘task-shifting’, is to facilitate delegation of tasks by one group of professionals to a less-scarce group, thus addressing difficulties with recruiting and retaining traditionally qualified staff.^22^^,^^23^ Maximising cost-effectiveness, by ensuring that tasks are done by the cheapest person who has the requisite skills, is a commonly mentioned secondary goal. This focus on negative drivers has been challenged by a European Expert Panel^24^ on task-shifting who emphasise the intervention’s potential to strengthen healthcare systems, placing *‘the best person* … *for the job’* in their article’s title.^24^ Several of their recommendations help situate our research, highlighting the need to consider effects on *‘the wider health system’* and to *‘engage in dialogue to understand the expectations and concerns of those who will be affected’* by task-shifting.^24^ Our participants’ dissatisfactions illustrate the need to make details of *‘increased workloads and responsibilities’* explicit and ensure that those taking them on *‘receive adequate remuneration’* for them.^24^

The expanding role of paramedics in doing GPs’ home visits was perceived by our participants as particularly salient. Although some mentioned tasks that paramedics did especially well, supporting the ‘best person for the job’ narrative, many nurses commented that paramedics were not ‘senior’ to themselves, and thus their advice was not an adequate substitute for ‘GP back-up’. The way that *‘a new professional group fits with other already established professions’* has been explored in relation to physician associates in general practice.^25^ Although this exploration was confined to effects on people working within a practice, the authors’ discussion of shifting boundaries and hierarchies between professionals is helpful for understanding similar shifts across the practice–community nursing interface: drawing on the notion of interdisciplinary boundaries as not simply fluid but dynamic,^26^ they make the distinction between vertical substitution, for example, where tasks are shifted from GPs to paramedics or nurses, and horizontal substitution, such as shifts between paramedics and nurses, and emphasise that normalising these changes depends on trust between different professional groups.

The ‘normal’ scope of nursing practice was discussed in several focus groups in our study. Although a few participants described the shifting and blurring of interprofessional boundaries as a normal phenomenon in itself, others spoke of being asked to take on tasks that they felt ‘should’ be done by a doctor. In particular, the work of assessing and managing a patient’s acute deterioration was associated with anxiety about ‘being wrong’. This anxiety mirrors evidence^27^ of caution among GPs about delegating responsibility to community nurses, underscoring the challenge of developing the interprofessional collaborations that are particularly important for delivering effective care in complex uncertain situations, such as those frequently encountered in palliative care.^28^

### Implications for research and practice

Our participants said they want ‘GP back-up’ with complex decision making in palliative care. Meanwhile GPs struggle with increasing workloads, and express concern about *‘the unclear role of primary care’* in end-of-life care.^29^ To clarify and shape that role, GPs and community nurses need to work together with policymakers, who should then fund the model agreed on and commission senior clinical ‘back-up’ from outside primary care if necessary.

More broadly, a recent list of priorities for research into service provision by GPs^30^ omitted any mention of community nurses. This omission should be rectified, acknowledging community nurses as key stakeholders in primary care research and policymaking, and drawing attention to the neglected but important interface between adjacent parts of what is supposed to be an integrated system.

In conclusion, GPs and community nurses collaborate within a complex ecosystem that cares for the increasing number of people who die at home. So changing the way GPs work inevitably affects community nurses. These effects deserve more attention than they have had so far.
