# Supplementary material for: How changes in GPs’ ways of working have affected community nurses: a qualitative study
Source: Br J Gen Pract. 2025 May 7;75(755):e406–11. doi: 10.3399/BJGP.2024.0534 (PMC12070296; doi:10.3399/BJGP.2024.0534)

Supplementary Box S1

Recruitment

Participants were recruited with the support of existing networks known to the authors and through the following organisations:
• Queen's Nursing Institute (QNI);<br>• Community Nursing Research Forum;<br>• Royal College of Nursing (RCN) District Nurse Forum;<br>• Royal College of Nursing (RCN) Pain and Palliative Care Forum;<br>• Hospice UK;<br>• Association of Palliative Medicine (APM);<br>• National Institute of Health Research Applied Research Collaboration (ARC);<br>• Associate of District Nurse and Community Nurse Educators (ADNE).
A link to the study webpage and expression of interest form was shared through social media channels (e.g., Twitter /X, Facebook) and/or email to potentially interested clinicians who were:
• working at the time of recruitment at band 5 or in a more senior role, in a community nursing team in the UK;<br>• English speakers;<br>• aged 18 years old or over;<br>• and had provided palliative care to patients within the past three months.
All participants who completed the online expression of interest form giving their contact information and professional characteristics (clinical band and role, length of care work experience in the community) were then contacted either via email or phone to discuss the study and gain informed consent.
Those who then gave written consent to participate in the study were emailed a link to complete a ten-minute online e-survey via Qualtrics, and asked whether they would also consider taking part in a 60-minute focus group.

Supplementary Table S1

Number of focus group participants in each category

Employment band:	Band 5	11
	Band 6	11
	Band 7	12
	Band 8	1
Years of community experience:	Up to 5 years	6
	6-15 years	19
	Over 15 years	10
Country of work:	England	31
	Scotland	3
	Northern Ireland	1

<u>Supplementary Information S1</u>

Semi-structured topic guide

Topic 1. How have things changed (15 minutes):

1) How have things changed for you, and those you work, with in providing end-of-life care since the onset of the pandemic?

- *Ask for examples to illustrate points made – could you explain a little more about what that has looked like in practice?*

2) Some doctors and nurses reduced their face-to-face patient contacts during the pandemic, using home or video contacts instead. How has this affected your own work with patients approaching the end of life?

- *Ask for examples to illustrate points made – could you explain a little more about what that has looked like in practice?*

Topic 2. Effects of taking on extended clinical roles (15 minutes):

3) How do you feel changes in your clinical roles / working practices have influenced patient and family care at the end of life?

- *Ask for examples to illustrate points made – could you explain a little more about what has helped patients' and families experiences, what has not helped, and why?*
- ***Explore both positive and negative impacts***

4) How do you feel changes in your clinical roles regarding end-of-life care have affected the way you work with colleagues, including general practitioner and specialists?

- *Ask for examples to illustrate points made – could you explain a little more about why this has helped or been a hinderance?* ***Explore positive and negative effects.***
- *Have more remote ways working and communicating with colleagues had an influence?*

Topic 3. Development opportunities (15 minutes):

5) Looking to the future, which changes in ways of practicing would you like to keep?

6) Are there some changes you think should be reversed?

7) Do you feel you and your community nursing team have the training and resources needed to take on extended clinical roles in end-of-life care? If not, what additional resources and training is needed to make this sustainable?

- *Prompt. Who is best placed to support improvements / the consolidation of skills?*

Close (5 minutes):

- **Go around the group asking if they have other comments they wish to make**
- **Thank group for participating. Re-iterate confidentiality**

Supplementary Figure S1

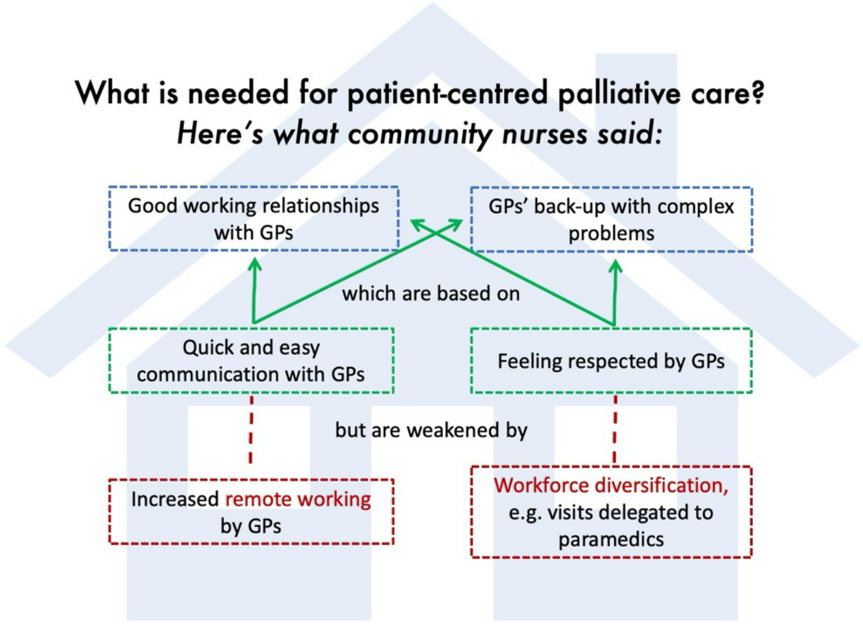

Supplement: Supplementary file 1 [file BJGP.2024.0534_suppl.pdf]
